# 

**Published:** 1876-05

**Authors:** 


CONTENTS.

| | Page |
|---|---|
| Family Order | 193 |
| New Potato | 196 |
| Human Growth | 196 |
| Poisonous Milk | 197 |
| Throat and Lungs | 200 |
| Toe Nails Inverted | 202 |
| Waste and Want | 202 |
| Divining Rods | 209 |
| How to Get Sick | 210 |
| Fly Exterminator | 212 |
| How a Doctor Looks | 213 |
| The Watchful Mother | 213 |
| An Easy Death | 214 |
| Insanity | 215 |
| Wearing Flannel | 217 |
| How to Prevent Colds | 218 |
| Curious Table of Figures | 219 |
| In a Hurry | 221 |
| Medical Principles | 221 |
| Nelaton's Suppository | 223 |
| The Panacea | 224 |
| Inverted Toe Nails | 224 |
| Our Country | 226 |
| Miasm and Malaria | 227 |
| Dentistry | 228 |
| Politeness | 228 |
| The Last Wish | 229 |
| The Air We Breathe | 229 |
| Sympathy for the Erring | 230 |
| True Temperance | 231 |
| Stomach and Liver | 232 |
| Dying Nations | 234 |
| Infants and Air | 234 |
| Leaning on Providence | 235 |
| Time Table | 236 |

